# Can Evidential Pluralism mitigate bias and motivated reasoning?

**DOI:** 10.1007/s11229-026-05530-z

**Published:** 2026-03-31

**Authors:** Joe Jones, Alexandra Trofimov, Michael Wilde, Jon Williamson

**Affiliations:** 1 King’s College London, London, UK; 2 University of Manchester, Manchester, UK; 3 Kent and Medway Medical School, Canterbury, UK

**Keywords:** Evidential Pluralism, Evaluation, Evidence-based medicine, Evidence-based policy, Bias, Effectiveness

## Abstract

This paper defends Evidential Pluralism, a philosophical account of causal enquiry, against the concern that it is particularly prone to bias and motivated reasoning. Evidential Pluralism scrutinises mechanistic studies alongside the comparative studies considered by the evaluation methods at the heart of orthodox evidence-based medicine and evidence-based policy. Concerns have been raised that mechanistic studies, and therefore Evidential Pluralism itself, are particularly prone to bias. We present a range of considerations to show that this is not the case.

Dissatisfaction with the rigid evidence hierarchies of orthodox evidence-based evaluation methods, such as those of evidence-based medicine (EBM) and evidence-based policy (EBP), have led to the development of more pluralistic approaches to evidence evaluation. But do these pluralistic approaches risk reintroducing the biased, motivated evidence-gathering practices that EBM and EBP were intended to replace? In this paper, we defend Evidential Pluralism, a specific philosophical account of causal enquiry, against such concerns. Evidential Pluralism broadens the evidence base by considering mechanistic studies alongside the comparative association studies that are the focus of orthodox evaluation methods, leading to approaches to evaluation called ‘EBM+’ and ‘EBP+’ respectively. We show that these new evaluation methods can mitigate some of the biases inherent in orthodox methods and can also mitigate biases that are specific to mechanistic studies and their evaluation.

We begin in Sect.  1 by explaining concerns about bias and motivated reasoning in orthodox evaluation methods. In Sect.  2, we then introduce Evidential Pluralism and explain how this alternative approach might lead to further concerns about bias and motivated reasoning. In Sect.  3 and subsequent sections, we show that these concerns are largely unfounded by explaining the ways in which Evidential Pluralism can mitigate bias: (i) the opportunity for cherry-picking and motivated reasoning is reduced by including all relevant studies in an evidence review, rather than a small selection of experimental studies; (ii) broadening the evidence base can reduce the influence of the biases inherent in any one study; (iii) association studies and mechanistic studies are prone to different and independent kinds of bias, which also helps to reduce the influence of each kind of bias; (iv) risk-of-bias analyses can be applied to mechanistic studies, just as they can to association studies (see Sect.  4); (v) Evidential Pluralism raises the evidential threshold for judging that effectiveness is established, which can lead to more robust and replicable judgements; (vi) Evidential Pluralism evaluations can be *structured* in ways that can further help to reduce bias and ensure replicable judgements (see Sect.  5); (vii) including mechanistic studies in the evidence base can eliminate prejudicial biases against stakeholder and qualitative evidence that are inherent in orthodox review. We illustrate several of these points by discussing a proof-of-concept Evidential Pluralism review of a legal intervention, namely face-mask mandates to reduce the spread of respiratory infections (Sect.  6).

## The potential for bias in orthodox evaluation

Evidence-based medicine (EBM) is famously defined as ‘the conscientious, explicit, and judicious use of current best evidence in making decisions about the care of individual patients’ (Sackett et al., 1996). Of course, few would disagree that medical decisions should be based on current best evidence. The question is: what counts as the ‘current best evidence’? Guyatt et al. say that:

Evidence-based medicine de-emphasizes intuition, unsystematic clinical experience, and pathophysiologic rationale as sufficient grounds for clinical decision making and stresses the examination of evidence from clinical research. (1992)

In other words, EBM maintains that the current best evidence is provided by the results of comparative clinical studies, such as randomized controlled trials, rather than unsystematic clinical experience, the intuition of experts, or mechanistic reasoning (aka ‘pathophysiologic rationale’). This conception of the ‘current best evidence’ soon caught on in other areas (Shan & Williamson, 2023, pp. 43–45). For example, evidence-based policy (EBP) has been defined as:

[T]he application of rigorous research methods, particularly randomized controlled trials (RCTs), to build credible evidence about “what works” to improve the condition; and the use of such evidence to focus public and private resources on programs, practices, and treatments (“interventions”) shown to be effective (Baron, 2018, p. 40).

It was an effective branding technique: few decision-makers, whether they were making medical or policy decisions, were willing to admit that their decisions were not based on the current best evidence. But what motivated this particular conception of the ‘current best evidence’? Why think that randomized controlled trials provide better evidence than mechanistic reasoning?

EBM was reacting to a different way of grounding medical decisions: so-called ‘eminence-based medicine’, where medical decisions were based on expert judgement. Proponents of EBM were concerned that eminence-based medicine was leading to ineffective or harmful medical decisions (Howick, 2011b, pp. 10–23). It was clear that eminence-based medicine was prone to bias. Of course, experts are human, and susceptible to biases and motivated thinking. But their expert judgements were often also based on their clinical experience, and clinical experience of a small number of patients can struggle to detect small differences, leading to false conclusions about the ineffectiveness of a medical intervention, that is, false negatives (Howick, 2011b, pp. 158–183). Clinical experience is also subject to the *post hoc ergo propter hoc* fallacy, which is a fallacy where one infers that events which occur sequentially are thereby causally related. For example, consider this sequence of events: the clinician prescribes some intervention; the patient recovers. A clinician might thereby infer that the prescribed intervention caused the patient’s recovery. However, such an inference could lead to false conclusions about the effectiveness of the intervention, that is, false positives (Worrall, 2007, p. 1001). Perhaps the patient would have recovered regardless. Clinical experience can confuse mere correlation with causation.^1^

Moreover, expert judgement was sometimes the result of ‘pathophysiological rationale’ or mechanistic reasoning, and mechanistic reasoning is itself prone to biases. Here is Miriam Solomon:

A general problem with mechanistic accounts is that they are typically incomplete, although they often give an illusion of a complete, often linear, narrative. Incompleteness is the consequence of there being mechanisms underlying mechanisms, mechanisms inserted into mechanisms, background mechanisms that can fill out the mechanistic story, and mechanisms that can hijack regular mechanisms. That is, there is complex interaction of multiple mechanisms in a chaotic and multidimensional system. There are possible hidden mechanisms everywhere in mechanistic stories, despite an easy impression of narrative or causal completeness. Since we do not have a theory of everything, it is not possible to know in advance whether or not a particular mechanistic intervention will have the intended result. (Solomon, 2015, pp. 131–132.)

In other words, even *experts* may too easily wrongly think they have a full understanding of the mechanisms responsible for health and disease. And when a mechanism is not fully understood, an intervention may wrongly seem ineffective when it is effective, or may wrongly seem effective when it is ineffective. Indeed, Jeremy Howick gives some examples of ‘cases where mechanistic reasoning led to the adoption of therapies that were either useless or harmful according to well-conducted clinical research’ (Howick, 2011b, pp. 154–157).

On the other hand, comparative clinical studies are thought to be at less risk of such biases. In particular, a large enough comparative study can detect the presence or absence of small effect sizes much better than clinical experience; they are thus safer from the biases associated with clinical experience (Howick, 2011b, pp. 158–183). Moreover, they can detect the presence or absence of small effect sizes without requiring anything much in terms of knowledge of the mechanisms of health and disease; comparative studies are thus safe from the biases associated with mechanistic reasoning (Ashcroft, 2004).

**Fig. 1:**
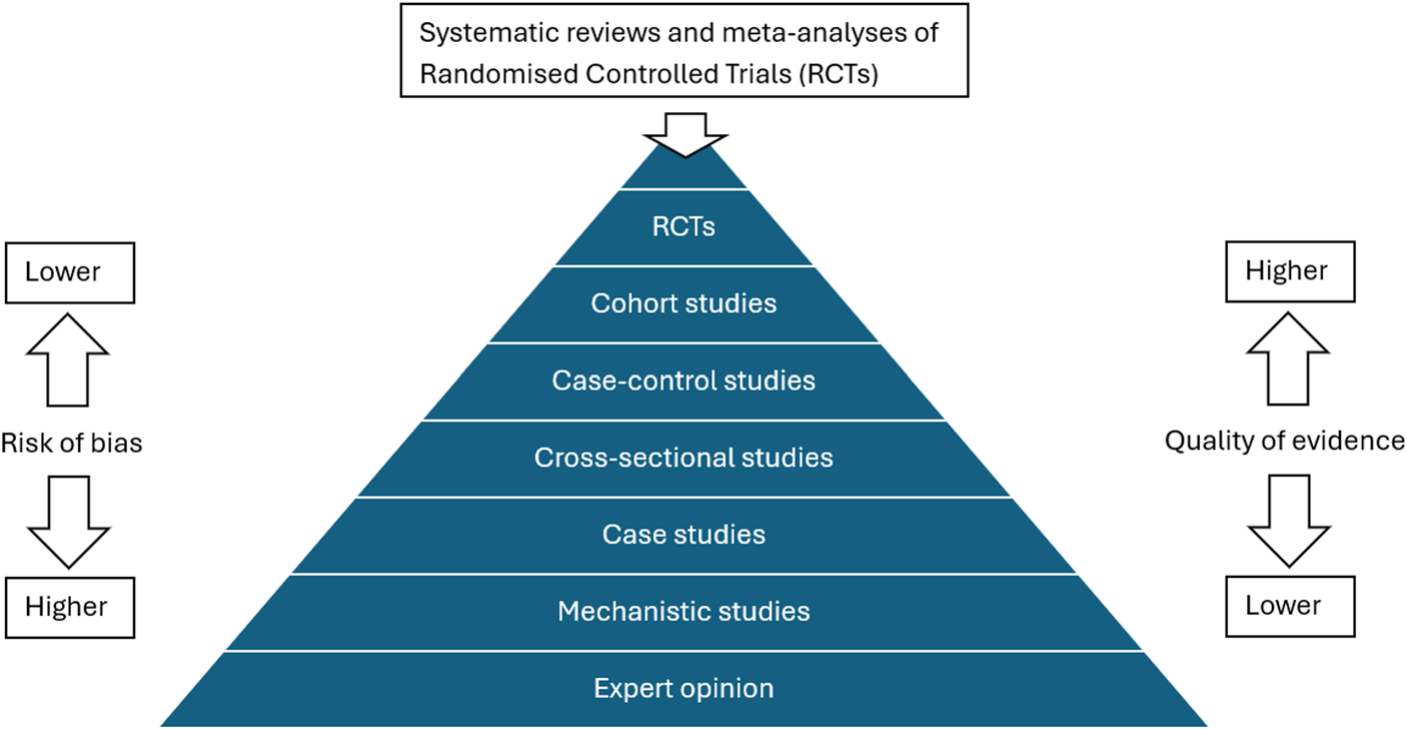
An evidence hierarchy, adapted from Yetley et al. (2017, p. 259S)

Of course, there are also biases that affect comparative clinical studies, such as confirmation, selection, and analysis biases. But there are methodological safeguards that attempt to address such biases: for example, confirmation biases are alleviated through double-blinding; selection bias is alleviated through randomization;^2^ and analysis biases are alleviated through pre-registration of studies (Stegenga, 2018, pp. 154–159). But studies can also be small and short: when a comparative study is small it may not have the power to detect a difference between the treatment and control arms of the study; and when a study is short, the trial may not detect the long-term effects of the treatment (Gillies, 2018, pp. 150–162). And then there is publication bias. Many comparative clinical studies are funded by pharmaceutical companies with a vested interest in publishing positive rather than negative results. It is thought best then to do a systematic review or meta-analysis of trials. This leads to a hierarchy of evidence, which ranks the risk of bias, and therefore quality, of different evidence-generating methods (Fig. 1).

Such a hierarchy of evidence is a proposed *ceteris paribus* ranking of evidence-generating methods: all other things being equal, a randomized trial is at less risk of bias than an observational study, such as a cohort study. However, it is rare that all other things are equal (Stegenga, 2018, pp. 71–83). For example: an observational study may have a much larger sample than a randomized trial; one randomized trial can be randomized or double-blinded better than another randomized trial; and one systematic review may be more comprehensive than another systematic review. Risk-of-bias tools have therefore been developed as a more nuanced way to assess the quality of the particular studies, by assessing the extent to which the different biases are present in those studies. But typically, such tools focus on assessing the risk of bias of comparative studies rather than mechanistic studies (see, e.g., Higgins et al., 2011). Perhaps this is because mechanistic studies are thought to be at such great risk of bias that a more nuanced assessment is not considered worthwhile.

There are remaining concerns about bias in relation to the orthodox approach. Firstly, the prioritisation of comparative studies, especially RCTs, risks skewing the research agenda towards questions and concerns that can be addressed by such studies. For example, as Khosrowi and Reiss (2019) argue, although high quality RCTs can provide good evidence of average treatment effects, they are of little use when distributive goals are pursued. A welfare policy, for instance, might aim at improving the welfare of the worst off. Although sub-group analysis or other study designs could provide the necessary evidence, they can be dismissed as inadequate due to the prioritisation of RCTs in the orthodox approach. Thus, ‘what kinds of policies are, or can be, justified’ is skewed because the orthodox approach ‘can systematically bias what the evidence can be used for’ (Khosrowi & Reiss, 2019, pp. 184-5).

Secondly, the orthodox approach has created an influential concept or stereotype of ‘good evidence’ that can result in biased dismissals of valuable evidence, including evidence of stakeholder perspectives and experiences, and these dismissals constitute epistemic injustice (Michaels, 2021; Mormina, 2022; Trofimov & Williamson, 2026). Consider, for example, the controversy surrounding Covid-19 public face mask mandates. It is argued that the extent to which comparative studies, especially RCTs, were prioritised and other kinds of studies were dismissed goes beyond reasonable scientific disagreement about what constitutes good evidence and is instead indicative of prejudice (Trofimov & Williamson, 2026). When evidence is prejudicially downgraded or dismissed, epistemic injustice is committed against the researchers and participants of the studies.

## Concerns about the potential for bias with Evidential Pluralism

As we have seen, orthodox evidence-based evaluation methods tend to focus on certain study designs—particularly RCTs—to the exclusion of other study designs, largely on the grounds that the favoured study designs, towards the top of the evidence hierarchy, tend to be less prone to bias than those further down. But is it correct to suggest that mechanistic studies are of lower quality than comparative studies, and that they have higher risk of bias? Evidential Pluralism would say not.

### What is Evidential Pluralism?

Evidential Pluralism is a philosophical theory of causal enquiry. Russo and Williamson (2007) put forward the core idea behind Evidential Pluralism, and the theory has subsequently been developed in the contexts of medicine (Parkkinen et al., 2018), the social sciences and policy evaluation (Shan & Williamson, 2023) and law (Trofimov & Williamson, 2025).

Evidential Pluralism can be motivated as follows. It is a platitude that *correlation is not causation*. This is because an observed correlation between two variables *A* and *B* of interest could be attributable to any of a wide variety of potential explanations:

*Causation*. *A* is a cause of *B*.

*Other causal explanations*. Reverse causation, confounding, confirmation bias, selection bias, analysis bias, ….

*Statistical explanations*. Chance, fishing, temporal trends.

*Non-causal connections.* Semantic, constitutive, mereological, logical, nomological or mathematical relationships between *A* and *B*.

**Fig. 2:**
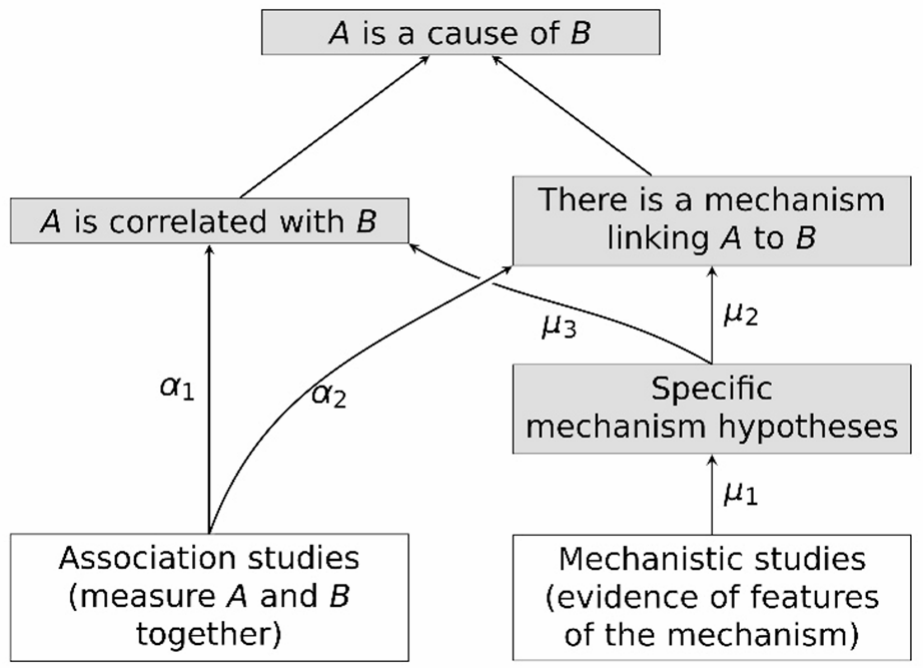
The evidential relationships posited by Evidential Pluralism (Williamson, 2021a)

Now, if it is indeed the case that *A* is a cause of *B*, then there must be some mechanism of action by which *A* brings about *B.* Furthermore, the mechanism complex as whole that links *A* to *B* (including counteracting, enhancing and enabling mechanisms as well as the mechanism of action) should be able to explain the extent of the observed correlation. So, to establish causation we need to establish both the existence of an appropriate correlation and the existence of an appropriate mechanism complex that can account for the correlation, as depicted in Fig. 2.

The usual way to test for a correlation is to perform a large comparative association study that repeatedly looks for *B* in the presence and absence of *A*, while controlling for potential confounding variables (channel α_1_ in the diagram). RCTs are particularly prized because they reduce the probability that the correlation is attributable to unforeseen confounding, indicating that it may be attributable to an underlying mechanism of action (α_2_). But there is a more direct way to confirm the presence of an appropriate mechanism: hypothesise key features of the mechanism (e.g., mediating variables, entities, activities and organisational structure), and perform studies that test for the presence of these features (µ_1_, µ_2_). In certain circumstances, these features can also support or undermine the claim that *A* and *B* are genuinely correlated (µ_3_).

Evidential Pluralism, represented by the above diagram, thus embodies two kinds of pluralism:

*Object pluralism*: a causal claim is established by establishing both correlation and mechanism.

*Study pluralism*: a causal claim is evaluated by evaluating relevant association studies and mechanistic studies.

Evidential Pluralism leads to a new approach to evidence-based medicine, called EBM+ (Parkkinen et al., 2018), a new approach to evidence-based policy, called EBP+ (Shan & Williamson, 2023), and a new approach to evidence-based law, called EBL+ (Trofimov & Williamson, 2025). These new approaches add the ability to scrutinise mechanistic studies to the methods of orthodox EBM, EBP and EBL.

### Concerns about bias

There are various concerns one might have about the potential for bias with Evidential Pluralism. Here we shall articulate some of these concerns, responding to them in subsequent sections.

First, one might worry that by scrutinising mechanistic studies alongside the association studies considered by orthodox evaluation, we are simply adding studies into the mix that have particularly high risk of bias. This view might be motivated by evidence hierarchies, such as that of Fig. 1, in which mechanistic studies are classified as having high risk of bias. The concern is that including such studies threatens to increase the influence of bias on judgements of overall effectiveness.

A second, related worry is that by scrutinising mechanistic studies, Evidential Pluralism is watering down the evidence base by including studies of inherently low quality. The suspicion is that decisions will be made on the basis of worse evidence. Again, this worry is motivated by evidence hierarchies such as that of Fig. 1. Howick et al. (2013), p. 275, have several concerns about the quality of mechanistic evidence, for example:

First, our understanding of mechanisms is often (and arguably, likely to remain) incomplete. Secondly, knowledge of mechanisms is not always applicable outside the tightly controlled laboratory conditions in which it is gained. Thirdly, mechanisms can behave paradoxically. Fourthly, as Daniel Steel points out, using mechanistic knowledge faces the problem of the ‘extrapolator’s circle’.

We encountered the incompleteness problem in the previous section. Howick (2011a, b) argues that incompleteness leads to poor judgements of effectiveness – e.g., mistakenly judging that interventions such as bloodletting, placing babies to sleep on their stomachs, and antiarrhythmic drugs were effective. Howick also points out that it is hard to predict how a mechanism will behave under intervention – that medicines can produce so-called ‘paradoxical’ responses, for example. Broadbent (2011), p. 56, expresses similar concerns about any appeal to mechanistic evidence in a method for causal inference:

Vandenbroucke’s argument stumbles on exactly the point I am trying to make, confusing the discovery of mechanism as a goal of causal inference, with the discovery of mechanism as method. I am suggesting that it is a good goal, but a lousy method.

Likewise, Sung and Holman (2023) observe that the drug aducanumab was given accelerated approval by the U.S. Food and Drug Administration (FDA) largely on the basis of mechanistic evidence, but they note that the evidence base seems to be weak in this case. The drug was subsequently abandoned.

A third worry is that Evidential Pluralism requires more judgements than orthodox evaluation does, and this increases the opportunity for bias, subjectivity and motivated reasoning to influence the evaluation of overall effectiveness. As we can see from Fig. 2, the Evidential Pluralism approach to evaluating effectiveness requires assessing the truth of various specific mechanism hypotheses, a correlation claim (that the intervention and outcome are correlated, conditional on potential confounders), a general mechanistic claim (that there exists some mechanism by which the intervention is responsible for the outcome and that can account for the magnitude of the observed correlation), and the causal claim (that the intervention is a cause of the outcome of interest). Orthodox evaluation does not seem to require so many judgements.

Andreoletti and Teira (2019) express this kind of concern. They distinguish *rules* (e.g., if there are two positive RCTs, approve the drug) and *standards* (e.g., standards for establishing effectiveness). They point out that ‘rules are less discretionary and, therefore, more difficult to game than standards’ (p. 1105), while ‘standard-based deliberation is, in itself, costly, as compared to a rule-based system, and, a priori, it is more difficult to protect against external pressures.’ (p. 1106). Orthodox evaluation methods seem to invoke simple rules, while Evidential Pluralism requires standards in order to reach judgements of effectiveness, and the suspicion is that standards are more malleable than rules.

A fourth concern is that Evidential Pluralism may be particularly prone to financial conflicts of interest. Thus Howick (2019) claims:

there are historical reasons to believe that emphasizing the evidential role of mechanisms may in fact exacerbate the problem with finance bias, because this view plays into the hands of industry. (p. 160)

the EBM+ proposal is entirely silent on the issue of financial conflicts of interest. … By ignoring the problem, they cannot possibly solve it. (p. 178)

The concern seems to be that by considering observational and mechanistic studies, pharmaceutical companies will have more opportunity to manipulate the evidence to favour their drugs.

Such a position would certainly have supported the view that antiarrhythmic drugs could be approved based on a surrogate endpoint, and as such, their proposals are certainly music to the ears of industry, in whose interest it is to circumvent the EBM standards that slow down the process of getting new treatments approved. (Howick, 2019, pp. 178-9)

Similarly, Holman (2019) argues that EBM + is a ‘friction-free’ account of medicine, in that it abstracts away from worldly complications to focus on the structure of inference, and that this sort of approach neglects the influence of the pharmaceutical industry and financial conflicts of interest, which are important worldly complications. Landes and Auker-Howlett (2024) express a similar concern.

We see then that there are two main kinds of concern about the Evidential Pluralism approach to evaluation: the suspicion that mechanistic evidence has high risk of bias and is of low quality, and the concern that EBM + and EBP+ require several judgements that might make the process open to manipulation and the influence of financial bias.

## Ways in which EP can mitigate bias

In this section we discuss how Evidential Pluralism mitigates the kinds of bias outlined above, developing certain proposals in more detail in subsequent sections.

Let us begin with the concern that mechanistic studies have high risk of bias and are of low quality. This is simply a category error: mechanistic studies can have a range of different study designs (including RCTs, observational studies, modelling studies and qualitative studies, for example) and these different designs are prone to different biases, some more substantial than others. It is thus non-sensical to categorise mechanistic studies alongside a range of particular study designs and to say that mechanistic studies have higher risk of bias and lower quality than these other study designs, as in Fig. 1.

Of course, because mechanistic studies can have a wide variety of study designs and some designs are more prone to bias than others, mechanistic studies *can* have high risk of bias. But biases attributable to study design can be mitigated by assessing such biases and discounting the studies that are more afflicted by bias. As we shall see in Sect.  4, risk of bias analyses can be applied to mechanistic studies, just as they can to association studies. Thus, individual studies, whether association or mechanistic, can be evaluated for their risk of bias, and the weight they are given in an Evidential Pluralism evaluation can take this risk of bias into account.

Furthermore, Evidential Pluralism’s requirement to scrutinise all relevant studies reduces the opportunity for motivated reasoning or cherry-picking evidence to support a particular aim or perspective. To ensure that an Evidential Pluralism review does not miss relevant evidence, various measures can be adopted. One can, for example, employ a systematic search like those conducted by orthodox systematic reviews, or a panel of experts and an open call for evidence, as is done by International Agency for Research on Cancer (IARC) evaluations, as we shall see in Sect.  5.

The requirement to scrutinise all relevant evidence also addresses the remaining concerns about bias in relation to the orthodox approach set out at the end of Sect.  1. By advocating a more inclusive approach to evidence, Evidential Pluralism avoids the research agenda being skewed towards problems and interventions that are more easily assessed using RCTs. Furthermore, by not committing to pre-determined hierarchies or rankings of evidence, Evidential Pluralism avoids creating a stereotype of good evidence that can lead to the prejudicial dismissal of non-comparative studies and thereby avoids the epistemic injustices that can result from the orthodox approach (Trofimov & Williamson, 2026).

Finally, broadening the evidence base can mitigate the influence of bias from individual studies. Increasing the quantity of evidence can reduce the influence of the biases of any individual study (Williamson, 2021b). The basic idea is that including more studies in an evaluation dilutes the influence of any individual study, and its biases, on the conclusion of the evaluation. If increasing the quantity of evidence did not help to reduce the influence of the biases of individual studies, then there would be little advantage to gathering more evidence. However, gathering more evidence is widely accepted as helpful for increasing confidence in the status of a causal claim.

Of course, increasing the quantity of evidence is unlikely to be very effective at controlling for bias if we simply have more of the same studies with the same kinds of bias. However, by including mechanistic studies alongside association studies, Evidential Pluralism increases the diversity of evidence which in turn can reduce the influence of bias from individual studies (Williamson, 2021b).

Association studies typically include, for example, RCTs, quasi-experimental studies and cohort studies. While these study designs can also serve as mechanistic studies, the range of mechanistic study designs is much broader and includes modelling studies, animal experiments and qualitative studies, for example. As we noted above, these study designs are susceptible to different sets of biases. Including a range of studies with different and independent biases can help to reduce the influence of the biases of individual studies. The idea is that different studies with different and independent biases can serve as a useful check on each other; if their results all point in a similar direction, then this can increase confidence in those results (Heesen et al., 2019). Thus, by considering mechanistic studies alongside association studies, Evidential Pluralism helps to reduce the influence of the biases to which association study designs are susceptible. In that sense, association studies and mechanistic studies act as independent witnesses (Williamson 2021b, p. 204). Note that mechanistic studies may also be less prone to financial conflicts of interest than association studies because they are often more likely to be free of industry funding (Gillies, 2019; Fugh-Berman, 2013).

Including association and mechanistic studies in an evaluation not only protects against bias by increasing the diversity of evidence but also through their reinforcing nature (Williamson 2021b; Clarke et al. 2014). Association studies are usually not sufficient on their own to establish causality. In particular, bias can play a key role in reducing the reliability of association studies as indicators of causality. By helping to determine whether an observed correlation is in fact attributable to a mechanism of action, mechanistic studies serve as a check on bias in association studies. Similarly, association studies can serve as a check on bias in mechanistic studies. By demonstrating a net association across the mechanism complex, association studies provide evidence that the putative cause does make a difference to the putative effect and that any unforeseen counteracting mechanisms do not negate the mechanism of action. The mutually reinforcing nature of association studies and mechanistic studies can therefore be understood as further reducing the risk of bias. As Williamson (2021b) puts it, ‘association studies and mechanistic studies are not fully independent witnesses: they are better than independent witnesses, because they make up for one another’s deficiencies’ (p. 204).

We now turn to the second main kind of concern facing Evidential Pluralism: the concern that there is an increased risk of bias as a result of Evidential Pluralism requiring more judgements.

Firstly, Evidential Pluralism raises the threshold for judging that effectiveness is established, which can lead to more robust and replicable judgements. According to Evidential Pluralism, establishing effectiveness requires not only high confidence in effectiveness but also high confidence that further research will not significantly reduce confidence in effectiveness (Parkkinen et al., 2018, p. 26). Orthodox evaluation, in contrast, draws non-numerical conclusions about effectiveness directly from quantitative estimates of ‘effect size’. Under the orthodox approach, an intervention is deemed effective if a confidence interval for average effect size does not include zero, even if it is plausible that further research will significantly change the estimate of effect size (Parkkinen et al., 2018 p. 26). By adding the requirement that there be high confidence that further research will not significantly reduce confidence in effectiveness, Evidential Pluralism raises the threshold for establishing effectiveness and helps to protect against the problem that estimates of effect size can be influenced by study bias. Judgements of effectiveness are therefore less susceptible to bias and more replicable.^3^

Secondly, Evidential Pluralism provides structured guidance on integrating different streams of evidence (Parkkinen et al., 2018). Providing structured guidance can help to protect against bias in judgements and ensure replicability. We shall provide an example of this in Sect.  5, where we consider IARC’s evaluation approach, which conforms closely to Evidential Pluralism and includes clear standards for making judgements of strength of evidence and a highly structured procedure for integrating subgroup assessments to reach an overall assessment of carcinogenicity. IARC also invokes strict rules and procedures to protect against financial and intellectual conflicts of interest. Procedures like this can be integrated within any Evidential Pluralism evaluation.

A further illustration of Evidential Pluralism’s bias mitigation measures is provided in Sect.  6 through a discussion of a proof-of-concept Evidential Pluralism evaluation of Covid-19 face mask mandates.

Bias will always be a concern. However, by enabling the systematic integration of diverse evidence streams, Evidential Pluralism provides greater protection against bias when compared to the orthodox approach to evaluation. There is, therefore, no reason to be especially concerned about bias in Evidential Pluralism.

## Risk-of-bias analyses applied to mechanistic studies

Mechanistic studies, like association studies, are prone to biases. The Oxford Centre for Evidence Based Medicine’s ‘Catalog of Bias’ (Nunan & Heneghan, 2025) lists over 65 different types of bias that are common in both mechanistic and association study designs, and outlines their individual risks, impact, and required preventative steps.

Some mechanistic studies use the same study designs as association studies, such as RCTs and observational study designs (Marchionni & Reijula, 2019). These studies, whether acting as mechanistic or association studies, will therefore be susceptible to biases induced by their design. For example, an RCT, whether acting as a mechanistic study or an association study, may be susceptible to ‘allocation bias’, where the allocation of participants is not properly randomised, thereby reducing the accuracy and reliability of the study. Any findings from this study may then be subject to further ‘observer bias’, where there are systematic discrepancies and inaccuracies in the process of observing and recording the results of a study, thereby allowing for conflicting, inaccurate, and unreliable interpretations. The risk of not addressing these biases is that the studies will not produce reliable or accurate results and will thus be less informative than they might otherwise be.

Mechanistic studies can also use study designs, such as agent-based modelling, that do not usually act as association studies and which are also prone to a range of biases.^4^ In some cases, these biases may be similar to, or the same as, biases arising in other study types. For example, any study that involves predictive modelling may be at risk of ‘confirmation bias’, if the modelling is conducted in such a way as to confirm the researcher’s own beliefs. Indeed, all studies are susceptible to ‘positive results bias’, which is the tendency to only publish positive results, and in turn the non-representation of any negative or null results. But modelling studies are also prone to distinctive kinds of bias, such as the biases induced by over-idealisation, or by a failure to check and validate the underlying modelling assumptions.

A well-recognised way to mitigate the influence of biases inherent in a study design is to use a risk-of-bias tool. One tool that is used widely by orthodox evaluation methods is the revised Cochrane risk-of-bias tool for randomized trails (RoB 2) (Sterne et al., 2019). Such tools can also be exploited by Evidential Pluralism: RoB 2, for instance, can used to assess the risk of bias of an RCT, whether it is acting as an association study or a mechanistic study. Tools for some other common study designs are listed in Table 1.

**Table 1:** Risk-of-bias tools for a range of common study designs

Study Design	Risk-of-Bias Tools
Randomised Control Trials (RCTs)	RoB 2: A revised Cochrane risk-of-bias tool for randomized trials (Sterne et al., 2019) Inclusion of a table/diagram laying out the assumptions and biases in a RCT (Krauss, 2018)
Non-randomised/quasi-experimental trials	ROBINS-I V2 tool (Sterne & Higgins, 2025) JBI Checklist for Quasi-Experimental Studies (Non-randomised experimental studies) (JBI, 2017a)
Cohort studies	Addressing of selection bias via directed acyclic graphs (Nohr & Liew, 2018) JBI Checklist for Cohort Studies (JBI, 2017b)
Ecological studies	ROBITT tool (Boyd et al., 2022) CEE Critical Appraisal tool (Konno et al., 2021)
In vitro studies	OHAT tool (NTP, 2015) SciRAP tool (Beronius, 2025)
Case-control studies	ROBINS-E tool (Higgins et al., 2024) Newcastle-Ottawa Scale (Wells, 2015)
Cross-sectional studies	CASP Cross-Sectional Checklist (CASP, 2024) JBI Checklist for Prevalence Studies (JBI, 2017b)
Systematic reviews	ROB-ME tool (Page et al., 2023) JBI Checklist for Systematic Reviews (JBI, 2017b)
Qualitative studies	Cochrane-Campbell Handbook for Qualitative Evidence Synthesis (Cochrane Qualitative and Implementation Methods Group, 2024) Confidence in the Evidence from Reviews of Qualitative research (Lewin et al., 2015)

If a risk-of-bias tool is not already available for a particular study design, a new risk-of-bias tool can be created and validated, as outlined below.

### Creating new risk-of-bias tools

Some mechanistic evidence may be drawn from study designs that do not have existing risk-of-bias tools, and it may be necessary for evaluators to create new risk-of-bias tools. Consider risk-of-bias tools for study designs that involve modelling. Tools like PROBAST (Wolff et al., 2019) and PROBAST + AI (Moons et al., 2025) have been developed to assess the risk of bias in prediction models and algorithms (and the systematic reviews thereof), by means of targeted questions developed through expert consensus. These are important, widely used tools, but they do not cover all kinds of modelling studies, nor all kinds of biases that affect modelling studies. For example, in agent-based modelling studies, these tools are important to assess the “participants and data sources, predictors, outcome, and analysis” (Moons et al., 2025, p. 1), but they do not address the biases underpinning the study designer’s rationale for the modelled agent’s actions. Thus, agent-based modelling may require a wholly new risk-of-bias tool.

When developing an entirely new risk-of-bias tool, it is often possible to use a similar approach to an existing tool. For example, RoB 2 employs an algorithm for identifying risk that is based on the answers to a questionnaire filled out by the reviewer, and a similar approach could be taken when developing a tool for agent-based modelling studies. Reviewers of modelling studies would be asked a series of questions regarding the rationale behind and functioning of the models used in the studies, with the answers they provide being used to identify the risk of bias. Considerations for such a tool might include:

- Does the study provide a clear justification of the theoretical assumptions and underpinning the agent-based model? Does it justify the idealisations employed by the model?
- Does the study provide a clear empirical or theoretical validation of the parameters of the agent-based model? Is the data used in any empirical validation process fully representative of the population of interest? How has missing data been handled?
- Are the advertised results of the study within the scope of the model, as validated? Does the study test the sensitivity of its results to small changes in the model idealisations or parameters?

### Validating new risk-of-bias tools

It is essential that any new risk-of-bias tool is properly validated before it is used, to ensure that it is effective in mitigating the influence of the biases in question and that its use is replicable across different raters. Common approaches to validating a risk-of-bias tool include the use of consultative expertise, such as the various iterations of the Delphi methodology (Nasa et al., 2021). The Delphi methodology is a collection of variations of a structured process for achieving expert consensus through the iterative questioning and feedback of an anonymised expert panel, often used in healthcare and policy development. When developing a new risk-of-bias tool, it is important to clearly outline the scope and intent of the tool in line with an established framework or underpinning piece of research. Many such frameworks already exist (Frampton et al., 2022). Where they don’t, other frameworks can be adapted, such as the quality of evidence rubrics proposed by Aston and Apgar (2023), which could be adapted into a bias rubric. Experts in the field can then be consulted on regarding the effectiveness of the proposed tool, and any feedback can be used to iterate the original proposal, until a version of the tool is reached that satisfies the consulting experts and study designers.

The responses from the experts themselves can also be assessed for inter-rater reliability by comparing the use of the tool by multiple members of the review panel, or by additional external experts. This acts as a calibration exercise to reduce bias in the consultation phase. While there are issues with the utilisation of expert consensus, it remains a vital additional step in verifying risk-of-bias tools: it applies the same diligence in identifying and addressing bias in the newly created tools as is applied when using the tools themselves to assess bias in primary studies (Minas & Jorm, 2010). A recent example of this process is provided by Fox et al. (2024), who identified the need for a risk-of-bias tool for assessing validity within clinical evidence syntheses and developed a pilot of such a tool through expert consensus. This demonstrates both that new risk-of-bias tools can be created and that validation for risk-of-bias tools can be obtained through existing consensus practices.

In sum, then, risk-of-bias tools can help to mitigate the influence of biases inherent in a range of study designs, and can be exploited by Evidential Pluralism, much as they can by the orthodox approach to evaluation. This may require creating and validating risk-of-bias tools for new study designs, but this is a well understood process.

## Structural ways to mitigate bias and motivated thinking

We have seen that risk-of-bias tools can be developed for mechanistic studies, just as they have been developed for comparative studies. However, there remains a concern that biases may be introduced when *identifying* and *integrating* the varied evidence from mechanistic studies and comparative studies. For instance, one way to identify and integrate a variety of different types of evidence involves expert group judgement. But, as we have seen, expert judgement is susceptible to biases. How then can the total evidence be identified and weighed up in a way that mitigates such biases?

In this section, we outline the methods employed by the *Monographs* programme of the International Agency for Research on Cancer (IARC). We point out that these methods are one way of identifying and integrating a variety of evidence broadly in line with Evidential Pluralism. We then point out five structural ways that help these methods to mitigate bias: (i) comprehensiveness; (ii) dividing the labour; (iii) using a strict framework; (iv) guidance by review experts; (v) transparency. Lastly, we suggest that these structural features could help more generally to mitigate biases when identifying and integrating varied evidence for an Evidential Pluralism review.

The *Monographs* programme of IARC aims to classify environmental exposures into one of four groups: Carcinogenic to humans (Group 1); Probably carcinogenic to humans (Group 2A); Possibly carcinogenic to humans (Group 2B); Not classifiable (Group 3) (IARC, 2019). They arrive at a classification by bringing together a working group of experts to identify and review all the relevant evidence concerning an exposure’s potential carcinogenicity. In coming to a classification, the working group considers three streams of evidence: studies concerning the exposure and cancer in humans (typically epidemiological studies); studies concerning the exposure and cancer in experimental animals; and mechanistic studies. So, the *Monographs* programme carries out evaluations broadly in line with Evidential Pluralism, looking at both association studies and mechanistic studies (Williamson, 2019).

While some have criticised the *Monographs* programme as susceptible to bias, Pearce et al. (2015) have provided a robust response to these criticisms. For instance, some have expressed concern that the processes of the *Monographs* programme lend themselves towards false positives (Boffetta et al., 2008, 2009). Pearce et al. point out that the vast majority of exposures evaluated by the *Monographs* programme are not classified as established causes or even probable causes of cancer; they also point out that ‘[t]he use of information from a variety of study designs reduces the likelihood of false-positive evaluations because it is unlikely that the same biases will occur in multiple studies based on different populations under different study designs’ (2015, p. 211).

One initial worry is that a classification may not be based on all the relevant evidence. The *Monographs* programme therefore takes steps to ensure that the identification of studies is as comprehensive as possible. IARC itself performs an initial literature search of various relevant databases, while also issuing a public and open invitation for others to submit studies for inclusion (IARC, 2019, p. 9). Moreover, the first responsibility of working group members is to ascertain ‘that all appropriate studies have been identified and selected’ (IARC, 2019, p. 10).

Evaluating such varied studies requires a variety of expertise: for example, expertise in evaluating epidemiological studies as well as expertise in evaluating mechanistic studies. Moreover, an expert in evaluating epidemiological studies may not be an expert in evaluating mechanistic studies, and vice versa. One worry then is that biases are introduced when integrating such varied evidence, since no member of the working group will have the expertise needed to evaluate all the relevant studies.

The *Monographs* programme addresses this worry by *dividing the labour*, thus making sure that studies are evaluated by appropriate experts. A given working group involves a range of experts, for example, members with the expertise to evaluate the relevant epidemiological studies, as well as members with the expertise to evaluate the relevant mechanistic studies. Each working group is then divided into four subgroups (IARC, 2019, pp. 12–29). One subgroup characterizes the substance, for example, by identifying the exposure and pointing out where it is most likely to occur. The other three subgroups consider different streams of evidence. The second subgroup reviews the epidemiological studies. The third subgroup reviews the studies in experimental animals. A fourth subgroup reviews the relevant mechanistic evidence. This division of labour helps to ensure that members of the working group are not reviewing studies outside of their expertise, thereby mitigating biases that might otherwise be introduced.

After they have reviewed the relevant studies, the second and third subgroup each come to a judgement about whether their particular stream of evidence provides sufficient, limited, or inadequate evidence for the carcinogenicity of the exposure, or whether there is evidence suggesting lack of its carcinogenicity (IARC, 2019, pp. 31–33). And the fourth subgroup also comes to a judgement about whether there is strong, limited, or inadequate mechanistic evidence (IARC, 2019, pp. 33–35). After this, the subgroups convene in a plenary session to bring together the judgements concerning the different streams of evidence in order to come to an overall classification for the exposure (IARC, 2019, pp. 35–37).

One might worry that the way in which they aggregate the evidence introduces biases associated with the subjectivity of expert judgement. For example, particular experts may give undue weight to particular streams of evidence: some experts may be biased towards the mechanistic studies, giving them too much weight in the overall classification; some experts may be biased against the mechanistic studies, assigning them too little weight in the overall classification. The *Monographs* programme attempts to address this worry by appealing to a strict framework for integrating the streams of evidence (see Table 2). In effect, there is an algorithm for coming up with an overall classification; the inputs to the algorithm are the judgements concerning the strength of the evidence from the individual subgroups; the output is an overall classification. For example, if there was judged to be limited evidence for the carcinogenicity of the exposure in epidemiological studies, but sufficient evidence in experimental animals and strong mechanistic evidence, then the overall classification of the exposure is Group 1 (see row 2 of Table 2). If there is judged to be sufficient human evidence, then the overall classification of the exposure is also Group 1.^5^ This strict framework is intended to make the overall classification more replicable, thereby mitigating the biases associated with the subjectivity of expert judgement.

**Table 2:**
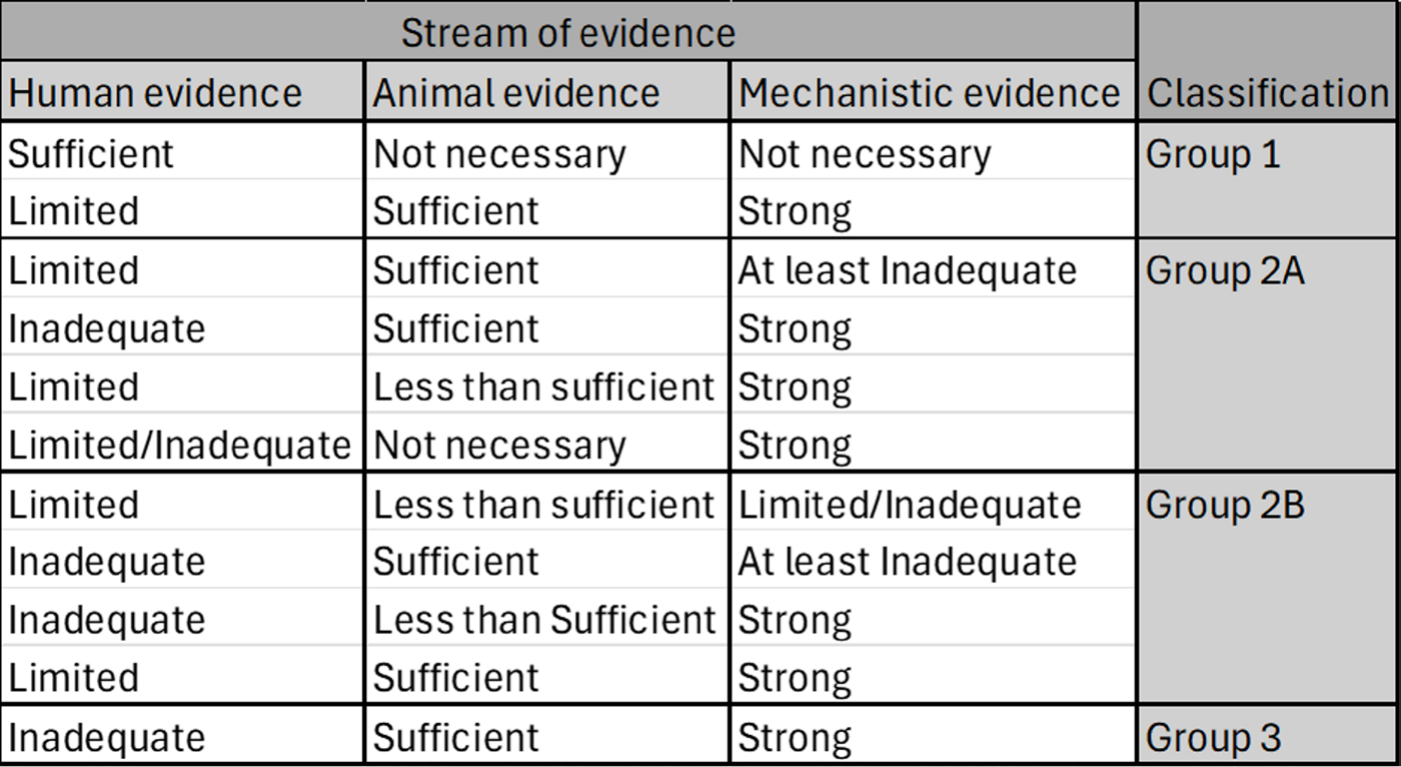
The strict framework for coming to an overall evaluation on the basis of the different streams of evidence, adapted from IARC (2019, p. 37)

But there is still room for biases to be introduced even in a strict framework. For instance, the members of a subgroup may not all share an understanding of what counts as ‘limited evidence’ from epidemiological studies, or ‘strong evidence’ from mechanistic studies; some members may set the bar inappropriately high; some inappropriately low. And even where there is shared understanding among members of a subgroup, this understanding may not be shared by subgroups from previous working groups. Again, the subjectivity of the experts here is another avenue for bias to be introduced. However, the *Monographs* programme addresses this worry by giving an important steering role to the IARC Secretariat. The IARC Secretariat are a group of review experts with particular expertise in the methods of the *Monographs* programme, which are described in the *Preamble* (IARC, 2019). They have typically worked for IARC for a number of years and have engaged with numerous previous working groups. So, they typically also have a good deal of relevant experience to draw upon. They are thus well-placed to clarify issues such as: ‘what counts as limited evidence from epidemiological studies?’ For instance, they could point to relevant sections of the *Preamble* for clarification (cf. IARC, 2019, p. 31). Or they could give examples of evidence that previous working groups judged to be ‘limited’, comparing and contrasting the evidence at hand. Again, the role of the Secretariat is to mitigate the biasing influence of the subjectivity associated with expert judgement by ensuring objectivity and consistency.

The worry might then arise that the Secretariat has an undue influence on the proceedings, thereby themselves introducing biases into the process. Again, the *Monographs* programme has a way of addressing such a worry: *transparency*. Members of the public and industry are welcome to observe proceedings to confirm that everything is above board. More generally, there are many opportunities for public and industry engagement with the process of a classification (IARC, 2019, p. 8). For example, IARC makes public beforehand the members of a working group and their declared conflicts of interest. This gives the public and industry an opportunity to scrutinise the list of members and report any undeclared conflicts of interest that may introduce bias into the proceedings (IARC, 2019, p. 6).

So, the *Monographs* programme has a number of structural ways for mitigating the biases that may be introduced by relying on expert judgement: (i) comprehensiveness; (ii) dividing the labour; (iii) using a strict framework; (iv) guidance by review experts; (v) transparency. Similar structural features can be exploited by an Evidential Pluralism evaluation involving expert group judgement. Indeed, the epistemic principles and procedures in Parkkinen et al. (2018) for an Evidential Pluralism review can be integrated with the structural features of the procedures at IARC, yielding a comprehensive framework for mitigating bias.

## Example: ways in which bias can be mitigated in a review of face-mask mandates

Greenhalgh et al. (2022) argue that during the Covid-19 pandemic, there was a narrow focus on RCT evidence to evaluate public health interventions, including public face mask mandates, and to inform decision-making. The narrow focus on RCTs had both epistemological and ethical consequences. Epistemologically, the focus on RCTs resulted in continued uncertainty regarding the effectiveness of face mask mandates. Ethically, the uncertainty led to delays in implementing a face mask mandate that potentially cost many lives (Greenhalgh et al., 2022).

Trofimov and Williamson (2025) conducted a proof-of-concept Evidential Pluralism evaluation of the effectiveness of Covid-19 face mask mandates in reducing the prevalence of symptomatic infections, hospitalisations and deaths. This example provides an illustration of how Evidential Pluralism can mitigate bias. Furthermore, it demonstrates that combining a diverse evidence base enables a robust and positive conclusion regarding the effectiveness of face mask mandates. Given Greenhalgh et al.’s (2022) criticism that the orthodox focus on RCTs led to problematic uncertainty and policy delays, it therefore provides a good example of the need for and benefits of an Evidential Pluralism evaluation.

The evaluation considered a wide and diverse evidence base, including RCTs, observational studies, modelling studies, surveys, event studies, focus group studies, natural experiments, regression models, ecological studies, human studies, experimental studies, systematic reviews and narrative reviews. All studies were assessed for their risk of bias by taking account of the biases acknowledged by study authors and by reviewing the literature for any additional concerns about bias. The weight they were given in the evaluation reflected this risk-of-bias assessment. The diversity of evidence helped to mitigate the influence of bias from individual studies on overall judgements.

In addition to including a diverse evidence base, the evaluation followed a systematic procedure. The evaluation was broken down into a problem claim, intervention claim, benefit claim and potential harm claim. For each of these causal claims, the correlation and mechanism claims were systematically assessed, and the diverse evidence was systematically integrated to reach an overall conclusion.

Consider, for example, the benefit claim. Here, the causal claim is that a legal requirement to wear a face mask in public reduces the prevalence of symptomatic SARS-CoV-2 infections, and thereby hospitalisations and deaths. The associated correlation claim is that a legal requirement to wear a face mask in public is negatively correlated with symptomatic infections, conditional on potential confounders. The mechanism hypothesis is that a legal requirement to wear a face mask in public increases face mask wearing which in turn decreases the prevalence of symptomatic SARS-CoV-2 infections, hospitalisations and deaths.

For the correlation claim, a range of association studies were considered. A sample of the studies considered is included in Table 3.

**Table 3:** Association studies in the proof-of-concept review of face mask mandates

Study	Study Conclusion	Risk of Bias
Adjodah et al. (2021) Event study covering 50 US States	Face mask mandates are associated with a decrease in infections, hospitalisations and deaths.	Study controlled for a number of confounders, but a risk of unexplained confounders remained.
An et al. (2021) Observational study covering 188 nations	Face mask mandates consistently associated with lower infection rates and mortality rates.	Controlled for a number of confounders, but not for enforcement or compliance
Lyu and Wehby (2020) Natural experiment covering 15 US States and Washington DC	Face mask mandates associated with a decline in infection growth rates.	Controlled for a number of confounders but not for enforcement or compliance.

Taken together, the association studies detected a robust correlation across contexts and controlled for a number of confounders. This made it unlikely that the correlation was spurious and therefore Trofimov and Williamson (2025) judged the correlation claim to be established. However, since a risk of residual confounding remained, a mechanistic evaluation could increase confidence in the causal claim. Trofimov and Williamson therefore judged it necessary to conduct a mechanistic evaluation.

To evaluate the hypothesised mechanism, a wide range of diverse studies were considered. A sample of the studies is included in Table 4.

**Table 4:** Mechanistic studies in the proof-of-concept review of face mask mandates

Study	Study Conclusion	Risk of Bias
Jefferson et al. (2023) Highly influential Cochrane systematic review. Included 18 RCTs/cluster trails.	Face masks make little to no difference to the spread of SARS-CoV-2.	Numerous design flaws. Most of the studies were not conducted during the Covid-19 pandemic and evaluated masks in terms of personal protection rather than source control. Studies were assessed for selection bias (random sequence generation / allocation concealment); performance bias (blinding of participants and personal); detection bias (blinding of outcome assessment); attrition bias (incomplete outcome data); reporting bias (selective reporting). Only one study rated as ‘low’ bias in all domains. Bar-Yam (2023) argue that the conclusions are biased towards the null as a result of the standard analytical equations used
Boulos et al. (2023) Rapid systematic review. Included 4 RCTs and 71 observational studies.	Face masks reduce transmission.	RCTs all rated high risk of bias. Observational studies mostly rated critical risk of bias, largely due to lack of controlling for other public health interventions.
Leffler et al. (2020) Ecological study / multivariate analysis of 196 countries.	Results support wearing masks to reduce the spread of SARS-CoV-2.	Potential for confounding at ecological level and information bias at individual and ecological level.
Van Doremalen et al. (2020) Comparative experimental study.	Results indicate that SARS-CoV-2 can remain infectious in the air for hours, supporting aerosol transmission.	Sampling of airborne virus technically challenging.
MacIntyre et al. (2021) Cross sectional surveys in Sydney and Melbourne (Australia), London (UK), and Pheonix and New York (USA).	Mandates identified as a predictor of mask wearing.	A risk of recall bias from the self-reported survey data and a risk of response bias from the recruitment method.
Puttock et al. (2022) Observational study covering 109,999 individuals in 126 US States.	Local mask mandates were associated with a 3-fold increase in mask wearing compared to no local mandate.	Study controlled for a number of confounders but there was a risk of some residual confounding, and the data may not have been fully representative.

Let us begin with the first part of the mechanism hypothesis: that a legal requirement to wear a face mask in public increases face mask wearing. Taking account of the diverse evidence, Trofimov and Williamson (2025) concluded that the first part of the mechanism hypothesis was provisionally established. All of the evidence considered supported the effectiveness of a legal requirement in increasing face mask wearing. Furthermore, the diverse studies had independent biases. For example, there was a risk of recall and response bias in the survey data in MacIntyre et al. (2021) whereas in Puttock et al. (2022) there was a risk of bias from residual confounding. The fact that the studies had independent biases increased confidence in the overall judgement of effectiveness: if studies with independent biases are all indicating that face mask mandates are effective at increasing face mask usage, this decreases the likelihood that the results are explained by bias. The studies, however, were not sufficiently representative of the global population. To increase confidence in the first part of the mechanism hypothesis, it would be necessary to find or conduct studies in a broader range of countries.

Now we turn to the second part of the mechanism hypothesis: that wearing a face mask helps to reduce transmission and thereby reduces infections, hospitalisations and deaths. On the basis of diverse evidence, Trofimov and Williamson (2025) concluded that the second part of the mechanism hypothesis was established. Let us consider this evidence.

The highly influential Cochrane Collaboration systematic review by Jefferson et al. (2023) found face masks make little to no difference to the spread of SARS-CoV-2. Given the numerous limitations of the studies included in the review, including a high risk of numerous biases, the results of the review should be taken as inconclusive rather than negative in relation to the effectiveness of face masks. Thus, while the review did not provide evidence to support the second part of the mechanism hypothesis, it also did not provide high-quality evidence against it.

All the other evidence considered supported the second part of the mechanism hypothesis. By extending the evidence base to include observational studies as well as RCTs, Boulos et al. (2023) reached a positive conclusion regarding the effectiveness of face masks. However, as Boulos et al. (2023) acknowledge, confidence in this conclusion should not be strong due to the high risk of bias in the observational studies. Incorporating a broader range of diverse evidence with independent biases enabled Trofimov and Williamson (2025) to reach a more confident conclusion that face masks reduce transmission and thereby symptomatic infections.

Trofimov and Williamson (2025) then systematically combined the evidence from association studies and mechanistic studies to reach an overall conclusion. The association studies detected a robust correlation across contexts. The strength of evidence of mechanisms increased confidence in the correlation claim to such an extent that further evidence would be unlikely to overturn it. The mechanistic studies provisionally established the first part of the mechanism hypothesis and established the second part of the mechanism hypothesis. Since a mechanism hypothesis can only be established to the extent of its weakest part, the overall mechanism hypothesis was deemed to be provisionally established. However, the strength of association studies substantially increased confidence in there being an underlying mechanism and therefore the general mechanism claim was established. Since both the correlation claim and the general mechanism claim were established, the causal claim was established. Thus, by systematically combining a diverse range of evidence from association studies and mechanistic studies, Trofimov and Williamson (2025) concluded that the benefit claim was established: that face mask mandates are effective at reducing SARS-CoV-2 symptomatic infections, hospitalisations and deaths.

This case study provides an illustration of some of the ways in which Evidential Pluralism can mitigate bias. Assessing individual studies for their risk of bias and including a diverse range of both association studies and mechanistic studies helped to reduce the risk of bias from individual studies. Furthermore, systematically assessing the correlation and mechanism claims and systematically integrating the evidence to reach an overall conclusion helped to reduce the influence of bias in judgements and ensure replicability.

As a proof-of-concept case study, however, it did not take advantage of all the bias mitigation measures available. A full Evidential Pluralism evaluation could further protect against bias in a number of ways. For example, measures could be taken to ensure all available evidence is considered, either by conducting a systematic search or an open call for evidence. Furthermore, risk-of-bias tools could be used to provide additional protection against individual study bias. Finally, incorporating the structural features of IARC evaluations explained in Sect.  5 could further protect against bias. Dividing the labour between subject experts and having review experts oversee the integration of evidence could further help to protect against bias in judgements while ensuring transparency could help to protect against potential conflicts of interest. Measures such as these are currently being used to conduct a more comprehensive mechanism-informed review of face-mask mandates (Greenhalgh et al., 2025).

This case study also provides an illustration of how the orthodox approach to evaluation can itself be charged with cherry-picking evidence. As we have seen in relation to Covid-19 face-mask mandates, the evidence from RCTs was inconclusive but the broader evidence base was strongly positive. By focusing only on RCTs, orthodox systematic reviews such as Jefferson et al. (2023) gave a distorted representation of the direction in which the evidence points. From the perspective of Evidential Pluralism, then, the orthodox focus on RCTs can be understood as cherry-picking evidence that points in a misleading direction.

## Conclusion

In this paper, we have attempted to alleviate any concern that Evidential Pluralism is particularly prone to bias. The influence of bias can be mitigated by the use of techniques exploited by existing approaches to evidence review, such as the use of risk-of-bias tools (Sect.  4) and a highly structured approach to review (Sect.  5). But Evidential Pluralism can also mitigate the influence of bias in ways that are not available to orthodox evidence review—by exploiting a broader evidence base, whose studies are prone to independent biases, for example (Sect.  3). IARC reviews (Sect.  5) and a proof-of-concept review of face-mask mandates (Sect.  6) lend confidence to the claim that, in practical use, bias is not an insurmountable problem for Evidential Pluralism.

Of course there is more to do to defend Evidential Pluralism. In particular, it would be interesting to show that, by generalising from a range of cases, the conclusions yielded by Evidential Pluralism are more reliable than those produced by orthodox methods. This seems plausible because Evidential Pluralism considers a strictly broader evidence base than orthodox methods and can thus avail itself of more information. If bias and motivated reasoning were to be a particular problem for Evidential Pluralism, that would undermine this consideration. But as we have seen, bias and motivated reasoning are not a particular problem. This leaves the path open for a case-based defence of Evidential Pluralism.

## Data Availability

Not applicable.

## Footnotes

1 Tabatabaei Ghomi and Stegenga (2025) give a more nuanced discussion of the conditions under which clinician’s causal inferences may be reliable.

2 For further discussion of the merits or otherwise of randomization, see, for example, Fuller (2019), Philippi (2022), and Maziarz (2025).

3 See Maziarz (2021) for further discussion of ways in which Evidential Pluralism can enhance replicability.

4 See Maziarz and Zach (2020) for discussion of the use of agent-based models as mechanistic studies.

5 Human evidence consists of epidemiological studies, so it might be objected that an exposure can be classed as a carcinogen based on evidence of correlation alone, and this in turn may look to go against Evidential Pluralism. However, this objection assumes that epidemiological studies provide only evidence of a correlation. Williamson (2019) argues that, under certain assumptions, epidemiological studies can also provide evidence of the existence of a mechanism.
